# CRISPR Typing Increases the Discriminatory Power of *Streptococcus agalactiae* Typing Methods

**DOI:** 10.3389/fmicb.2021.675597

**Published:** 2021-07-19

**Authors:** Clémence Beauruelle, Ludovic Treluyer, Adeline Pastuszka, Thierry Cochard, Clément Lier, Laurent Mereghetti, Philippe Glaser, Claire Poyart, Philippe Lanotte

**Affiliations:** ^1^Département de Bactériologie-Virologie, Hygiène Hospitalière et Parasitologie-Mycologie, Centre Hospitalier Régional Universitaire (CHRU) de Brest, Brest, France; ^2^Inserm, EFS, UMR 1078, GGB, Universitè de Bretagne Occidentale, Brest, France; ^3^Institut Cochin, Team Bacteria and Perinatality, INSERM U1016, Paris, France; ^4^INRAE, ISP, Université de Tours, Tours, France; ^5^Service de Bactériologie-Virologie, CHRU de Tours, Tours, France; ^6^Evolution and Ecology of Resistance to Antibiotics (EERA) Unit, Institut Pasteur, Paris, France; ^7^UMR CNRS 3525, Paris, France; ^8^CNRS UMR 8104, Paris Descartes University, Paris, France; ^9^Department of Bacteriology, University Hospitals Paris Centre-Cochin, Assistance Publique-Hôpitaux de Paris, Paris, France

**Keywords:** CRISPR-Cas, group B *Streptococcus*, *Streptococcus agalactiae*, typing, molecular subtyping

## Abstract

We explored the relevance of a Clustered regularly interspaced short palindromic repeats (CRISPR)-based genotyping tool for *Streptococcus agalactiae* typing and we compared this method to current molecular methods [multi locus sequence typing (MLST) and capsular typing]. To this effect, we developed two CRISPR marker schemes (using 94 or 25 markers, respectively). Among the 255 *S. agalactiae* isolates tested, 229 CRISPR profiles were obtained. The 94 and 25 markers made it possible to efficiently separate isolates with a high diversity index (0.9947 and 0.9267, respectively), highlighting a high discriminatory power, superior to that of both capsular typing and MLST (diversity index of 0.9017 for MLST). This method has the advantage of being correlated with MLST [through analysis of the terminal direct repeat (TDR) and ancestral spacers] and to possess a high discriminatory power (through analysis of the leader-end spacers recently acquired, which are the witnesses of genetic mobile elements encountered by the bacteria). Furthermore, this “one-shot” approach presents the benefit of much-reduced time and cost in comparison with MLST. On the basis of these data, we propose that this method could become a reference method for group B *Streptococcus* (GBS) typing.

## Introduction

*Streptococcus agalactiae* or group B *Streptococcus* (GBS) is the leading cause of neonatal infections and an emerging pathogen in adults, particularly in elderly and immunocompromised patients (Phares et al., 2008; Skoff et al., 2009; Slotved and Hoffmann, 2020; Vuillemin et al., 2021). It is also a commensal bacteria that colonize the gastrointestinal and genitourinary tracts of 10–30% of healthy humans (Regan et al., 1991; Campbell et al., 2000; Khalil et al., 2017; Cho et al., 2019). Prevention of GBS-related neonatal infections involves the detection of vaginal carriage in pregnant women, followed by intrapartum antibiotic prophylaxis for those colonized (ANAES, 2001; Schrag et al., 2002).

To characterize GBS isolates, two main typing methods are widely used: serotyping and MLST (multi locus sequence typing). Serotyping methods, which are based on differences in capsular polysaccharides (CPS), were first evaluated by phenotypic methods and then by PCR-based molecular methods (Imperi et al., 2010). To date, 10 serotypes (or capsular types) have been identified (Ia, Ib, and II-IX) (Cieslewicz et al., 2005; Slotved et al., 2007). The prevalence and distribution of serotypes are known to differ between geographical regions, ethnic populations, and clinical presentations (Schuchat, 1998; Kwatra et al., 2016). MLST, a sequence-based method, is now widely used to investigate the population structure and genetic lineage of GBS and is currently the reference method for GBS typing. This approach is based on the combination of alleles for seven housekeeping genes (Jones et al., 2003). Unique combinations of the alleles at each locus define the allelic profiles, or sequence types (STs). ST can be clustered in clonal complexes (CCs) when six of the seven alleles are in common (Feil et al., 2004). These methods have highlighted the involvement of serotype III and ST17, in causing more invasive neonate diseases (Manning et al., 2009). Among adults, serotype V isolates belonging to CC1 have been associated with invasive disease (Bergseng et al., 2008; Skoff et al., 2009). More recently, serotypes III, Ia, and IV have gained relevance in this context (Tazi et al., 2011; Lamagni et al., 2013; Teatero et al., 2014, 2015). Despite the clonality observed within GBS populations causing invasive disease, increasing diversity has appeared, hence the need to investigate the population structure. However, between these two typing methods, while serotyping is relatively easy to perform in the laboratory, it is insufficiently discriminating to compare isolates. MLST presents significant disadvantages: it is relatively costly, time-consuming, and labor-intensive. Moreover, as the sequences targeted evolve slowly, MLST is not highly discriminant for epidemiological studies and local surveillance, and isolates are not easy to distinguish at the ST level (Radtke et al., 2010; Haguenoer et al., 2011; Sabat et al., 2013).

For typing, whole genome sequencing (WGS) provides an ideal resolution and accuracy (Schürch et al., 2018; Kayansamruaj et al., 2019; Beauruelle et al., 2020; Perme et al., 2020). This method shows higher discriminatory power than capsular typing and MLST. However, although WGS is increasingly accessible, it is still an expensive technology requiring experience and skill to be used, including bioinformatics analysis. Indeed, WGS is not yet useful for epidemiological studies or local surveillance. An accessible typing method for GBS strains is definitely needed and useful at this moment, especially for low-to-middle-income country and in the view of the advanced development stage of GBS vaccines, which will require robust and continuous surveillance worldwide. At the same time, other approaches have been developed such as the analysis of patterns of virulence gene, prophages content, or CRISPR-Cas [Clustered regularly interspaced short palindromic repeats (CRISPR) and CRISPR associated sequences (Cas)] analysis (Van der Mee-Marquet et al., 2006; Lopez−Sanchez et al., 2012; Lier et al., 2015; Crestani et al., 2020).

CRISPR-Cas is an adaptive and vertically transmitted immune system present in a large proportion of prokaryotic genomes (Barrangou et al., 2007). CRISPR arrays are made up of a succession of highly conserved repeated sequences, called direct repeats or DRs, interspaced by sequences of a similar length, called spacers. Most CRISPR arrays are flanked on one side by the leader sequence and on the other side by a trailer sequence, which is preceded by the terminal direct repeat (TDR) of the CRISPR array, corresponding to a degenerated or truncated DR. DRs are highly conserved within a locus, whereas spacers vary widely as they derive from foreign genetic elements. In this bacterial immune system, each spacer is acquired in response to mobile genetic element (MGE) mostly considered as invasive elements by the bacteria. Spacers are integrated in a linear, time-oriented manner. Each new spacer is incorporated at the leader end of the CRISPR array, concomitantly to the duplication of a DR. Because of the polarized acquisition of spacers deriving from encounters with various MGE over time, CRISPR arrays constitute a chronological archive of past encounters. Thus, spacers located at the leader-end extremity are recently acquired and represent recent contacts with MGE, whereas spacers located at the trailer-end extremity represent ancestral spacers. Leader-end spacers enable the differentiation of closely related strains and trailer-end spacers reflect broader phylogenetic relationships. Indeed, several studies have shown potential for CRISPR-based typing (Barrangou and Dudley, 2016).

In GBS, two CRISPR-Cas systems have been characterized, a type I-C system named CRISPR2, which is rare and most often incomplete, suggesting little or no activity, and a type II-A system, named CRISPR1, which is ubiquitous and functional (Lopez−Sanchez et al., 2012). The CRISPR1 array is made up of highly conserved DR of 36 bp, separated by spacers of approximately 30 bp, which are extremely diverse in sequence and in number across GBS strains studied. Similarities between CRISPR1 spacer sequences and MGEs have been previously reported (Lopez−Sanchez et al., 2012; Lier et al., 2015). Comparative sequence analysis across numerous GBS isolates emphasized that CRISPR1 array is extremely diverse and evolves *in vivo*, demonstrating the dynamics of the system (Lopez−Sanchez et al., 2012; Lier et al., 2015; Beauruelle et al., 2017, 2018).

A typing method based on CRISPR array analysis has been shown to be a useful tool for comparing GBS isolates (Lopez−Sanchez et al., 2012; Lier et al., 2015; Beauruelle et al., 2017, 2018; Gajic et al., 2019). This method presents the advantage of being linked to the genetic lineage defined by MLST through analysis of the TDR and ancestral spacers. Moreover, exploration of the recent evolution of the isolate, especially encounters with MGE, is possible through the analysis of the leader-end spacers. Based on the spacers’ variability, it seems to be in congruence with MLST with a greater discriminating power. However, these advantages still have to be confirmed.

The aim of our study was to evaluate CRISPR1 analysis as a high-resolution *S. agalactiae* typing method and to compare this method to capsular typing and MLST.

## Materials and Methods

### Bacterial Isolates

Two hundred and fifty-five *S. agalactiae* isolates were analyzed. A total of 224 isolates were collected at the University Hospital of Tours, France, between 2002 and 2016; 26 were collected by the National Reference Center for *Streptococci* in 2012 and five were reference strains. Among the clinical isolates, 198 were isolated from adults and 52 were isolated from neonates. Among them, 164 were isolated in a non-invasive setting from cutaneous lesions (*n* = 2), urinary tract (*n* = 5), gastric aspirate (*n* = 7), respiratory tract (*n* = 9), genital tract (*n* = 119), or intestinal tract (*n* = 23), and 86 were isolated in an invasive setting from blood (*n* = 49), CSF (*n* = 33), or joint fluid cultures (*n* = 2). Isolates were grown on GBS selective media or blood agar plates. Identification was performed using MALDI-TOF MS (Vitek MS, BioMerieux France, or Bruker Daltonics Germany). The five reference strains considered were 2,603 V/R (capsular type V, ST110), A909 (capsular type Ia, ST7), NEM316 (capsular type III, ST23), COH1 (capsular type III, ST17), and BM110 (capsular type III, ST17) (Glaser et al., 2002; Tettelin et al., 2005; Da Cunha et al., 2014; Supplementary Table 1).

### DNA Extraction

Genomic DNA was extracted following enzymatic lysis with mutanolysin (Sigma). A bacterial suspension of 1.5 McFarland was prepared in 500 μl of water containing 50 U of mutanolysin. The suspension was incubated for 1 h at 56°C, followed by 10 min at 100°C, leading to cell lysis. Lysates were centrifuged for 3 min at 1,500 × *g* and the supernatants containing DNA were collected.

### Capsular Typing

Capsular typing was performed by PCR-based methods based on previously described methods (Imperi et al., 2010). The assay identified each serotype (Ia to IX) by analysis of band patterns on agarose gel.

### Multilocus Sequence Typing

Multi locus sequence typing was carried out as previously described (Jones et al., 2003). Allelic profiles and ST were assigned using the international MLST database^1^. CCs were defined using the stringent group definition (6/7 shared alleles) and eBURST analysis^2^ applied to the 250 isolates of the study.

### CRISPR1 Array Amplification and Sequencing

CRISPR1 array amplification was performed using CRISPR1 PCR-F and CRISPR1 PCR-R primers that target the CRISPR1-flanking regions as previously described (Lopez−Sanchez et al., 2012; Lier et al., 2015; Beauruelle et al., 2017). PCR products were sequenced using the internal sequencing primers CRISPR1 SEQ-F and CRISPR1 SEQ-R. For CRISPR1 regions exceeding 1.3 kb, primers targeting internal spacers were used to complete the sequencing. Primer sequences are presented in Supplementary Table 1.

### CRISPR1 Array Analysis

Spacers, repeats, and flanking regions for each sequence were identified using a macro-enabled Excel tool (P. Horvath, DuPont). This tool allows the identification and extraction of CRISPR features from nucleotide sequences, and the graphic representation of spacers as colored cells in Excel spreadsheets. The macro-enabled Excel tool is optional and sequence obtained could be analyzed without it by the identification of the DR sequences (Supplementary Table 2) separating each spacer sequence. Spacer sequences were compared to the dictionary of spacers established earlier and expanded previously (Lopez−Sanchez et al., 2012; Beauruelle et al., 2017, 2018). New spacers identified in this study further expanded the dictionary and were numbered incrementally. The original contributions presented in the study are publicly available. These data can be found at http://crispr.i2bc.paris-saclay.fr/CRISPRcompar/Dict/Dict.php.

### Spacers and TDR Selection

Graphic representation of CRISPR1 arrays made it possible to separate isolates according to their CRISPR1 array composition. Isolates were clustered according to their ancestral spacers and TDR composition. Among each cluster, specific markers, corresponding to spacers and TDR, were selected in view of separating isolates according to their CRISPR1 array similarity. Markers were selected according to their frequency among CRISPR array and their specificity to each phylogenetic group. Markers were first selected visually thanks to the macro-enabled Excel tool used. Markers selected were then analyzed using a binary code (Supplementary Table 3) to (i) evaluate the frequency of the marker selected among isolates and among each group, (ii) evaluate the absence of redundancy between markers selected, and (iii) evaluate the specificity of each marker for a group. Markers selected had to be widely present and specific of each CRISPR array belonging to a given phylogenetic group. Markers selected were present in at least three-quarters of the isolates of the group, were non-redundant, and were specific of each group considered. Our aim was to obtain the best compromise between marker number and discriminatory index (DI).

### Data Analysis

The DI described by Hunter and Gaston was used as a numerical index for the discriminatory power of each typing method (Hunter and Gaston, 1988). The categorical coefficient, unweighted pair group with arithmetic mean (UPGMA), and the minimum spanning tree (MST) were run using BioNumerics 7.6.2 software (Applied Maths, Sint-Martens-Latem, Belgium). In MST, each circle represents a CRISPR genotype or a CC and its size is proportional to the number of isolates. The thicker branches link the genotypes differing by only one spacer, the thinner branches link genotypes differing by more than one spacer. Congruence between CRISPR1 typing, MLST, and serotyping was calculated using Rand Index (BioNumerics software).

## Results

### Capsular Typing and Multi Locus Sequence Typing

#### Capsular Typing

Among the 255 *S. agalactiae* isolates analyzed, six capsular types were represented. The main capsular type was type III (*n* = 93), followed by type Ia (*n* = 56), V (*n* = 54), Ib (*n* = 23), II (*n* = 17), and IV (*n* = 12). Capsular types VI, VII, VIII, and IX were not found among our isolates (Table 1 and Supplementary Table 4).

**TABLE 1:** Characteristics of the 255 GBS isolates tested classified by sequence type.

ST	No. of isolates	Serotype (no. ofisolates)	Source (no. of isolates)*
ST1	32	II (2), V (30)	NIA (22), IA (9), IN (1)
ST2	5	Ia (1), Ib (1), II (1), IV (1), V (1)	NIA (5)
ST3	1	IV (1)	NIA (1)
ST4	3	Ia (2), II (1)	NIA (1), IA (2)
ST6	3	Ib (3)	NIA (2), IN (1)
ST7	2	Ia (1), V (1)	NIA (1), Ref.A909
ST8	12	Ib (11), V (1)	NIA (8), IA (4)
ST10	7	Ib (1), II (2), IV (1), V (3)	NIA (4), IA (2), IN (1)
ST12	7	Ia (1), Ib (4), II (1), III (1)	NIA (4), IA (3),
ST17	56	III (56)	NIA (18), IA (2), NIN (3), IN (31), Ref. COH1 – BM110
ST19	26	II (1), III (22), V (3)	NIA (18), IA (5), IN (3)
ST22	2	II (2)	NIA (2)
ST23	37	Ia (34), III (3)	NIA (22), IA (7), NIN (2), IN (5), Ref. NEM316
ST24	4	Ia (4)	NIA (3), IN (1)
ST26	3	V (3)	NIA (2), IA (1)
ST27	1	III (1)	NIA (1)
ST28	10	Ia (1), II (5), III (2), V (2)	NIA (8), NIN (2)
ST41	1	III (1)	NIA (1)
ST88	1	Ia (1)	NIA (1)
ST110	1	V (1)	Ref. (2,603 V/R)
ST130	3	Ia (2), V (1)	NIA (3)
ST136	1	IV (1)	NIA (1)
ST144	2	Ia (2)	NIA (2)
ST173	1	V (1)	IA (1)
ST182	1	III (1)	NIA (1)
ST196	8	Ia (1), Ib (1), IV (6)	NIA (7), IA (1)
ST220	2	Ia (2)	NIA (2)
ST223	1	Ia (1)	NIA (1)
ST243	1	Ia (1)	NIA (1)
ST255	1	Ib (1)	IA (1)
ST291	1	IV (1)	IA (1)
ST297	1	V (1)	NIA (1)
ST305	1	Ia (1)	NIA (1)
ST327	2	V (2)	NIA (2)
ST366	1	III (1)	NIA (1)
ST370	1	V (1)	NIA (1)
ST385	1	Ia (1)	IA (1)
ST386	1	II (1)	IA (1)
ST388	2	V (2)	NIA (2)
ST389	1	III (1)	NIA (1)
ST390	1	Ib (1)	NIA (1)
ST391	1	III (1)	NIA (1)
ST459	1	IV (1)	IA (1)
ST481	1	III (1)	IA (1)
ST569	1	II (1)	NIA (1)
ST1002	1	III (1)	NIA (1)
ST1004	1	III (1)	NIA (1)
ST1005	1	V (1)	NIA (1)

*NI, non-invasive isolates; I, invasive isolates; A, adult; N, neonate; Ref., reference strains.

#### MLST

A total of 48 different STs were found among the 255 isolates. The most common was ST17 (*n* = 56), followed by ST23 (*n* = 37), ST1 (*n* = 32), ST19 (*n* = 26), ST8 (*n* = 12), and ST28 (*n* = 10). Other STs were represented by fewer isolates (<10) (Table 1). Invasive isolates belonged mainly to ST17 followed by ST23 and ST1, while non-invasive isolates were distributed mainly among ST23, ST17, ST1, and ST19 isolates. eBURST analysis clustered the ST into seven CCs [CC17 (grouping ST17, ST291, and ST1004), CC1 (grouping ST1, ST196, ST2, ST1005, ST459, ST136, ST370, ST297, and ST173), CC23 (grouping ST23, ST220, ST1002, ST481, ST385, ST88, ST144, ST366, and ST391), CC19 (grouping ST19, ST28, ST27, ST389, ST182, and ST386), CC8 (grouping ST8, ST12, ST10, and ST390), CC6 (grouping ST6, ST7, ST255, and ST41), and CC4 (grouping ST4, ST3, and ST243)], one group with two singletons ST (ST26 and ST388), and five other singletons (ST22, ST24, ST130, ST327, and ST569) (Supplementary Table 5). All CCs except CC17 contained mainly non-invasive isolates [CC19 (*n* = 31, 77.5%), CC1 (*n* = 38, 74.5%), CC23 (*n* = 34, 70.8%), CC6 (*n* = 4, 66.7%), CC8 (*n* = 17, 63%), CC4 (*n* = 3, 60%), and CC17 (*n* = 22, 39.3%)] (Supplementary Table 5). Using UPGMA and MST, the 48 different profiles (corresponding to the 48 STs) were grouped into 21 clusters with more than one isolate (up to 54 isolates by cluster) (Supplementary Figure 5).

### CRISPR1 Array Analysis

**FIGURE 1:**
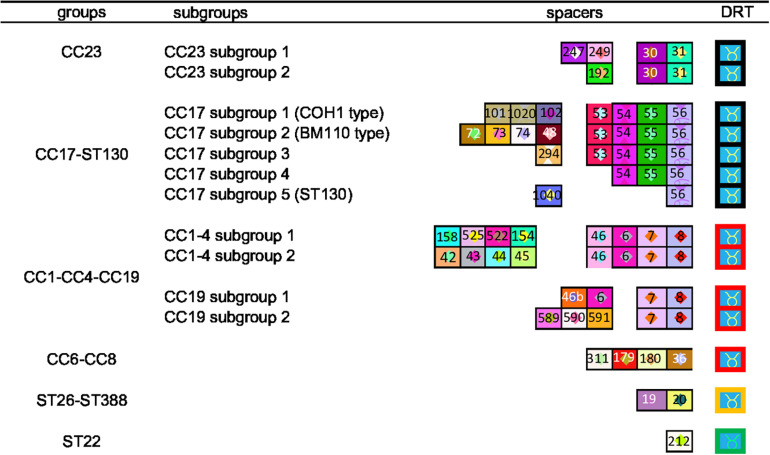
CRISPR clustering according to TDR and ancestral spacers. The CRISPR1 arrays are represented using a macro-enabled Excel tool, whereby spacers are converted into two-color symbols based on spacer sequence. Repeats are not shown except terminal direct repeats (TDRs), which are represented by different colored borders according to their sequence. Spacers were identified by a number attributed following the spacer dictionary (http://crispr.i2bc.paris-saclay.fr/CRISPRcompar/Dict/Dict.php). Arrays are oriented with respect to the leader sequence located on the left. A total of 14 clusters were obtained. The six groups were defined by TDR and ancestral spacers. These groups were then divided into subgroups, based on more recent spacers.

We generated a complete CRISPR1 sequence for all the 255 isolates. The number of spacers ranged from three to 29 per isolate, corresponding to a CRISPR array size of 266–1,800 bp. Among the 255 isolates, a specific CRISPR1 array was observed for 92% of isolates (*n* = 229). For the other 8%, the same CRISPR1 array was common for two isolates (*n* = 22) or for four isolates (*n* = 4). Using the macro-enabled Excel tool, isolates could be distributed according to their CRISPR1 array homology (Supplementary Figure 1). First, isolates were grouped into six clusters according to their TDR and ancestral spacers. These six clusters showed a correlation between CC defined by MLST and eBURST analysis: a CC23 cluster; a cluster grouping CC17 and ST130 isolates; a cluster grouping CC1, CC4, and CC19 isolates; a cluster grouping CC6 and CC8 isolates; a cluster grouping ST26–ST388 isolates; and a ST22 cluster (Figure 1). CRISPR1 clusters were then divided into subgroups according to more recently acquired spacers. In this way, a total of 14 clusters and subgroups were defined, namely, three subgroups in cluster CC1–CC4–CC19, allowing separation between CC19 (two subgroups) and the group CC1–CC4 (two subgroups), five subgroups in the CC17 cluster (COH1 type, BM110 type, ST130 type, and two other subgroups), and two subgroups in the CC23 cluster (Figure 1 and Supplementary Figure 1). This CRISPR genotyping approach can be used to rank some singletons defined by eBURST analysis into clusters: ST24 (CRISPR1 cluster CC23), ST130 (CRISPR1 cluster CC17), ST569 (CRISPR1 cluster CC6–CC8), and ST327 (CRISPR1 cluster CC19) (Supplementary Table 5 and Supplementary Figure 1).

**FIGURE 2:**
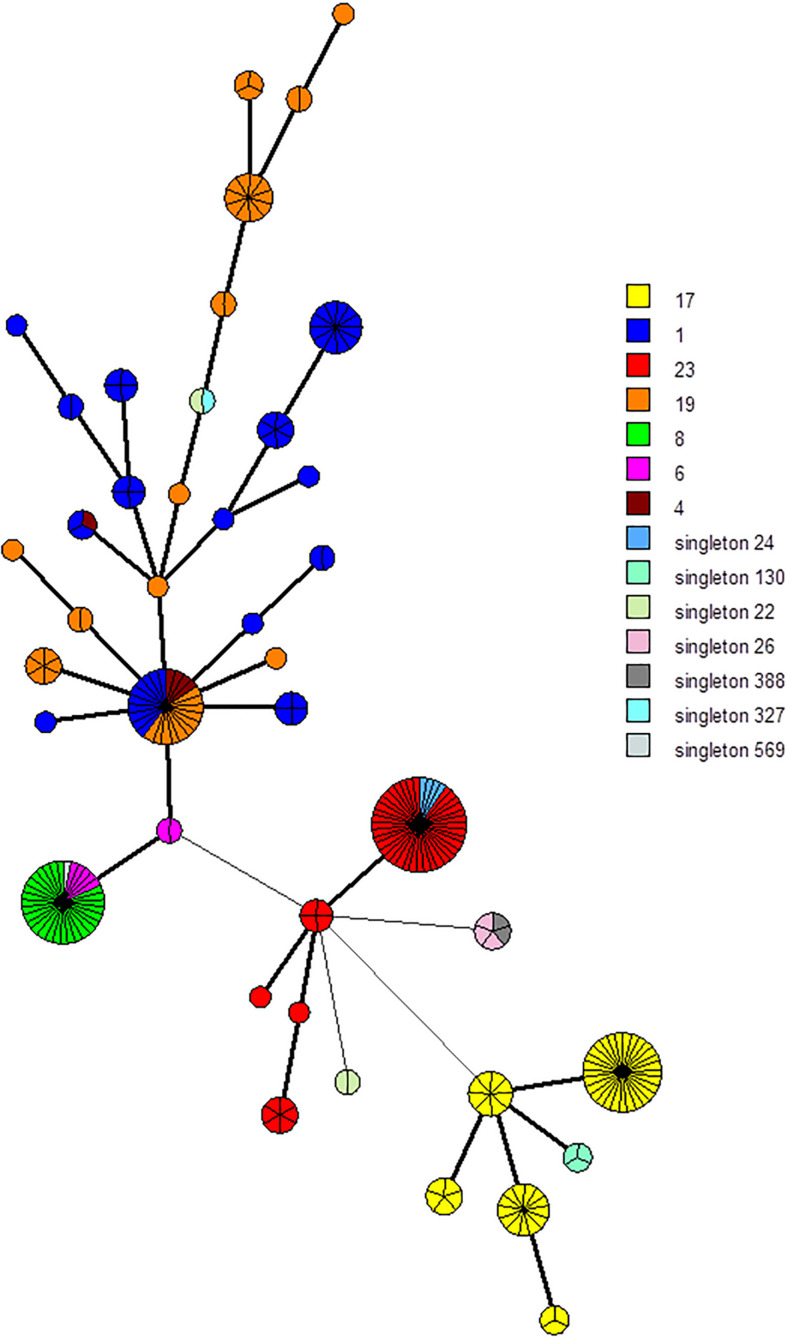
Minimum spanning tree (MST) representation of the 25 CRISPR1 markers scheme clustering. Each circle represents a CRISPR1 genotype and its size is proportional to the number of strains. Each color represents CC or singleton defined by MLST (e.g., yellow for CC17). A high level of correlation between this marker selection and MLST type was observed; circles (representing CRISPR1 genotype) are characterized mostly by a same color, especially for CC17 and CC23, whereas isolates belonging to CC1 and CC19 are more dispersed.

Among each cluster, spacers and TDR were selected to define phylogenetic lineages. Each spacer selected was cluster- and subgroup-specific. We selected two groups of markers. The first involved 94 markers, namely, the five different TDRs and 89 spacers (Supplementary Table 3A). The second involved 25 markers, namely, the five different TDRs and 20 spacers (Supplementary Table 3B). Among the 255 isolates, using the UPGMA algorithm, the 94-marker scheme defined a total of 172 different profiles and 45 groups with more than one isolate (up to eight isolates) (Supplementary Figures 3, 4). Likewise, the 25-marker selection defined a total of 42 different profiles and 29 clusters with more than one isolate (up to 40 isolates) (Figure 2 and Supplementary Figure 4). Using these two-marker selections, groups previously defined were divided in different clusters (Figure 2 and Supplementary Figure 2). Thus, CC17 isolates were divided into 30 groups and five groups, respectively, using these two-marker selections (94 and 25 markers, respectively).

### Differences Among Clusters

Some groups displayed lower spacer composition diversity. Among the 26 isolates sharing a mutual CRISPR1 profile, 17 belonged to CC17, and the nine others belonged to CC8 (*n* = 2), CC23 (*n* = 3), CC19 (*n* = 2), and ST388 (*n* = 2). Indeed, the majority of CRISPR1 arrays sharing a mutual profile belonged to CC17 isolates and represented 31% (17/54) of them. Among these CC17 isolates with common CRISPR1 array, the main isolates (76%, 13/17) belonged to the first CRISPR1 subgroup (COH1 type) and represented 41% of isolates of the COH1 type subgroup (13/32). By contrast, isolates belonging to CC1 and CC19 shared a high degree of polymorphism and could not be easily clustered into their specific CC defined by MLST and eBURST (Supplementary Figure 1).

### Discrimination of Isolates by CRISPR Analysis

CRISPR1 array analysis makes it possible to separate isolates within a same ST or capsular type. Among the 255 isolates, six capsular types and 48 different STs were observed, while CRISPR1 analysis separated them into 229 different CRISPR1 profiles, and the two marker schemes into 172 (94-marker scheme) and 42 (25-marker scheme) profiles. The diversity index was compared for the two CRISPR1 specific marker schemes as well as for MLST. The diversity index was 0.9947 for the CRISPR1-specific 94-marker scheme, 0.9267 for the CRISPR1-specific 25-marker scheme, and 0.9017 for the MLST method on this population (Table 2).

**TABLE 2:** Hunter and Gaston diversity index of CRISPR typing scheme with 94 markers or 25 markers and MLST.

Typing method		Diversity index
CRISPR GenoTyping	94 markers	0.9947
CRISPR GenoTyping	25 markers	0.9267
MLST		0.9017

*The two-marker group selection makes it possible to separate isolates with a high diversity index, superior to both MLST and capsular typing, highlighting the high discriminatory power of this CRISPR GenoTyping approach.*

### Congruence Between CRISPR1 Typing, MLST, and Capsular Typing

Congruence between the two CRISPR1-specific markers, MLST and capsular typing, was analyzed using the Rand Index (Supplementary Table 6). A strong congruence was observed between the 25-marker scheme and the 94-marker scheme (Rand Index: 0,932). The Rand Index between CRISPR1 marker selections and MLST was 0.904 and 0.902 for the 25-marker scheme and the 94-marker scheme, respectively. The Rand Index between CRISPR1 marker selections and capsular typing was 0.797 and 0.766 for the 25-marker scheme and the 94-marker scheme, respectively. The Rand Index between MLST and capsular typing was 0.84.

## Discussion

The aim of our study was to evaluate CRISPR1 analysis as a high-resolution *S. agalactiae* typing method. We explored the relevance of the CRISPR1-based genotyping tool, and we compared this method to current molecular standards. We developed two schemes of markers selection (94 or 25 selected markers corresponding to spacers and TDR), and we simultaneously characterized these isolates by MLST and capsular typing. We used a representative library of the species by selecting a wide variety of isolates that differed by their anatomic and geographic origin, as well as by their phylogenetic origin.

Markers were selected in view of clustered isolates according to their CRISPR1 array similarity. TDR and ancestral spacers allow the isolates to be linked to their common ancestor. More recent spacers allow separating isolates in the subgroup within this first selection. We defined two schemes of marker selection. The largest selection allowed the clustering of the majority of isolates. To facilitate data mining, we tried to reduce the number of markers as much as possible. A number of 25 markers appears sufficient to discriminate isolates successfully. The discriminatory power of this CRISPR1 approach is superior to that of both capsular typing and MLST (diversity index of 0.9947 and 0.9267 for the two marker schemes vs. 0.9017 for MLST). The CRISPR1-typing approach and the two-CRISPR1 marker selection, including the simplest, allow one to efficiently separate isolates and to successfully discriminate isolates that were considered indistinguishable by MLST. Furthermore, compared to publish data, the CRISPR1 discriminatory power appears superior to that of pulsed-field gel electrophoresis, which is considered highly discriminatory (Simpson’s diversity index = 0.92) (Pillai et al., 2009). Aside from its high discriminatory power, this CRISPR1 typing approach assesses the phylogenetic structure of the *S. agalactiae* population, with the two schemes of CRISPR1 marker selection showing a clonal distribution of the population similar to that obtained by MLST. The CRISPR1 clustering approach generated major clusters that corresponded well to the main CC obtained by MLST and eBURST analysis. Moreover, this approach presents the major advantage of being highly discriminant thanks to the variability of the CRISPR1 array. The high DI of the CRISPR1 approach made it possible to distinguish between isolates within the same ST or CC. This approach delivers a unique DNA fingerprint and makes it possible to separate isolates, even clonal bacteria such as *S. agalactiae*. A strong congruence was observed between the 25-marker scheme and the 94-marker scheme (Rand Index: 0.932), as well as between the two marker schemes and MLST (Rand Index: 0.904 and 0.902 for the 25-marker scheme and the 94-marker scheme, respectively). The congruence between CRISPR1 marker selection and capsular type was lower (0.797 and 0.766 for the 25-marker scheme and the 94-marker scheme, respectively). This could probably be explained by the high discriminatory power of the CRISPR typing technique compared to serotyping.

Previous studies highlight the interest of CRISPR for GBS typing (Lopez−Sanchez et al., 2012; Lier et al., 2015; Beauruelle et al., 2017, 2018; Gajic et al., 2019). In particular, these studies highlight the correlation between CRISPR and MLST and the strong discriminatory power of CRISPR typing. In the present study, we confirm the interest of GBS CRISPR typing by specifying the added value of this method compared to other commonly used, including those based on the calculation of diversity and congruence index. Moreover, we show that this discriminatory power of CRISPR1 typing is also applicable with a limited number of markers, offering a good compromise between discriminatory power, phylogenetic data, and simplicity.

We then challenged our marker selection with other groups of isolates based on CRISPR array publicly available from two publications (Lopez−Sanchez et al., 2012; Gajic et al., 2019). Gajic et al. (2019) characterized 87 CRISPR1 array from GBS isolates from Serbia (invasive and non-invasive human isolates). Following our spacer selection, all of the isolates from Gajic et al. (2019) study could be clustered in the groups and 90% of them in the subgroups. All except one (Serbie 41 isolate) could not be clustering using our two marker schemes. We also evaluated our spacer selection with the isolates from the Lopez−Sanchez et al. (2012). study. This publication presents the great advantage of including CRISPR1 array of GBS isolated from animals and from several geographic regions. Among the 351 GBS isolates, 87% (*n* = 306) could be clustered in the groups and 80% in the subgroups. Marker selections allow clustering in subgroups 97% and 86% of isolates with the 94- and 25-marker scheme, respectively. The 45 isolates, almost exclusively animal isolates, which could not be clustered in a group, belonged to CC340, CC260, CC61–67, and CC103. In our study, these CCs were not present and could not be classified using our marker selections. Further studies are needed to enlarge the diversity of GBS isolates studied to be able to classify isolates belonging to less frequent or peculiar lineages.

Within CRISPR1 locus analysis, CRISPR1 diversity differed among clusters. CC17 isolates shared a low degree of polymorphism compared to other CCs, as noted previously (Lier et al., 2015; Beauruelle et al., 2017). This moderate CRISPR1 diversity could be explained by the slow rate of evolution of ST17 isolates, characterized by a low rate of recombination, which is known for contributing to CRISPR array diversity (Da Cunha et al., 2014). Another explanation might be that CC17 isolates encode a specific repertoire of surface proteins, suggesting a specific colonization site, and therefore a genetic isolation (Da Cunha et al., 2014). Conversely, some CCs shared a high degree of polymorphism and were difficult to cluster using CRISPR1 array. This relates in particular to isolates belonging to CC1, CC4, and CC19. Interestingly, CC1 isolates are increasing in adult invasive infections since the 1990s, suggesting the evolution of this CC, especially ST1 isolates (Bergseng et al., 2008; Skoff et al., 2009). We can hypothesize that this CRISPR1 diversity could be due to (i) a more rapid evolution of these CC isolates, (ii) a more active CRISPR-Cas system, or (iii) a higher diversity of MGE attacking this CC leading to enhanced CRISPR immunization of this phylogenetic lineage. Nevertheless, lineages defined by CRISPR1 analysis, including in less variable CCs, correspond to different lineages previously highlighted by WGS, which is undoubtedly the gold standard to compare isolates (Da Cunha et al., 2014). Indeed, according to TDR and ancestral spacers, CC17 isolates could be divided into four subgroups and CC23 isolates could be divided into two subgroups (Figure 1) as previously described by SNP analysis (Da Cunha et al., 2014). Furthermore, WGS has previously confirmed CRISPR1-based clustering for the *S. agalactiae* population (Kayansamruaj et al., 2019; Beauruelle et al., 2020). Conversely, some CCs shared a high degree of polymorphism and were difficult to cluster using CRISPR1 array. This relates in particular to isolates belonging to CC1, CC4, and CC19, suggesting a greater heterogeneity of this population. This may be due to the high recombination rate in some CCs as previously described (Da Cunha et al., 2014).

CRISPR1 array analysis is easy to perform with just one array of limited length (266 to 1,800 bp) to be analyzed (unlike MLST, which requires seven loci to be sequenced) and offered supplementary information than that obtained by MLST and capsular typing combined. Moreover, this “one-shot” approach generates data (spacers dictionary) that enable comparison of isolates between different laboratories. Considering these data, CRISPR1 typing appears as an *ad hoc* tool to compare isolates and analyzed GBS transmission. Nowadays, although incidence of late onset neonatal GBS disease and adult GBS disease is increasing (Bekker et al., 2014; Ballard et al., 2016), their transmission routes are poorly understood. This CRISPR1 based approach could be a useful tool to explore source of transmission of these GBS infections. Moreover, this approach has the ability to assess the genetic relatedness among these isolates and to provide a better understanding of the physiopathology of these infections. Whereas the analysis of ancestral spacer and TDR allows typing, the analysis of spacers at the leader end (recently acquired spacers) allows subtyping and provides specific evidence on the recent evolution of isolates, especially encounters with MGEs. MGEs are key factors for the evolution of bacteria, including for GBS as highlighted by the insertion of integrative and conjugative elements that caused the expansion of few clones in human, particularly adapted to their host (Da Cunha et al., 2014). Indeed, following GBS–MGEs contact, CRISPR analysis gives us valuable clues. Similarly, GBS possesses a broad animal host spectrum and studies proved that some GBS genotypes can cause human invasive diseases through animal sources as food-borne zoonotic infections (Tan et al., 2016). A deep phylogenetic analysis such as CRISPR typing appears useful to analyze the circulation of different GBS genotypes in humans and animals in different countries and to monitor potential emerging zoonotic GBS clones. CRISPR1-based typing could be used to explore the genetic relatedness among humans and animal isolates such as cattle and fish, which are also an important source of GBS infection (Radtke et al., 2010; Liu et al., 2016). In the present work, we selected a wide variety of human isolates but we did not analyze animal isolates, which is one limitation of this work.

Another interest to this CRISPR1-based typing method was the clinical evaluation of GBS vaccine. The advanced development stage of GBS vaccine requires robust and continuous surveillance worldwide, including in low-income countries. This CRISPR approach has already proven its effectiveness to evaluate evolution and diversity of GBS vaginal carriage (Beauruelle et al., 2017, 2018). Indeed, this low-cost and easy-to-use method appears useful to evaluate the diversity of the species, including in low-income countries where there is little information available about the GBS isolate characteristics. The majority of vaccine development was based on polysaccharide conjugate vaccine, and evaluation of GBS diversity is usually based on the capsular type (Berner, 2021; World Health Organization, 2021). However, serotype replacement should be kept in mind (Meehan et al., 2014). Moreover, GBS vaccine efficacy on different GBS genotypes as well as GBS carriage evolution over time (same or new isolate) could be evaluated with this tool.

Although WGS is increasingly accessible, it is still an expensive technology requiring experience and skill to be used, including bioinformatics analysis. Nowadays, whole-genome sequencing still does not appear to be a routine method for genotyping, thus rendering CRISPR-based typing technology useful during the transition from the current molecular typing method to the omic level. Given these data, we assume that this method could become an actual reference method for phylogenetic GBS typing.

## Data Availability Statement

The original contributions presented in the study are publicly available. This data can be found here: http://crispr.i2bc.paris-saclay.fr/CRISPRcompar/Dict/Dict.php.

## Author Contributions

CB and PL designed the study, developed methods, and wrote the manuscript. CB, LT, CL, PG, and CP selected bacterial isolates and performed the research. TC analyzed data and performed bioinformatic analysis. AP and LM provided critical feedback of the manuscript. All authors analyzed data, contributed to manuscript revision, read, and approved the submitted version.

## Conflict of Interest

The authors declare that the research was conducted in the absence of any commercial or financial relationships that could be construed as a potential conflict of interest.

## Footnotes

1 http://pubmlst.org/sagalactiae/

2 http://eburst.mlst.net/v3/mlst_datasets/
