# Supplementary material for: CRISPR Typing Increases the Discriminatory Power of Streptococcus agalactiae Typing Methods
Source: Front Microbiol. 2021 Jul 19;12:675597. doi: 10.3389/fmicb.2021.675597 (PMC8328194; doi:10.3389/fmicb.2021.675597)
Supplement: Supplementary file 1 [file Data_Sheet_1.pdf]

Supplementary Material

Isolates	CC	ST	capsular type	leader
Tours-S291	23	23	Ia	gag
Tours-C230	23	144	Ia	gag
Tours-C75	23	305	Ia	gag
Tours-C133	23	220	Ia	gag
Tours-S12	23	23	Ia	gag
Tours-S322	23	23	Ia	gag
Tours-S102	23	23	Ia	gag
Tours-C52	23	223	Ia	gag
Tours-S148	23	23	Ia	gag
Tours-S125	23	23	Ia	gag
Tours-S158	23	23	Ia	gag
Tours-Sag14	23	23	Ia	gag
Tours-Sag505	23	23	Ia	gag
Tours-S108	23	23	Ia	gag
Tours-S101	23	23	Ia	gag
Tours-Sag232	23	23	Ia	gag
Tours-Sag313	23	23	Ia	gag
Tours-Sag94	23	23	Ia	gag
Tours-C57	23	23	Ia	gag
Tours-C18	23	23	Ia	gag
Tours-P1E4	23	23	Ia	gag
Tours-Sag387	23	23	Ia	gag
Tours-Sag288	23	23	Ia	gag
Tours-Sag468	23	23	Ia	gag
Tours-S.ag434	23	23	Ia	gag
Tours-S318	23	23	Ia	gag
Tours-S264	23	23	Ia	gag
Tours-P5A2	23	23	Ia	gag
Tours-C196	23	23	Ia	aaa
Tours-E198	23	385	Ia	gag
Tours-Sag328	23	23	Ia	gag
Tours-S.ag8	23	23	Ia	gag
Tours-S.ag 416	23	23	Ia	gag
Tours-S.ag152	23	23	Ia	gag
Tours-C20	23	23	Ia	gag
Tours-S.ag183	23	144	Ia	gag
Tours-Sag22	23	23	Ia	gag
Tours-Sag4	23	23	Ia	gag
Tours-S167	Singleton	24	Ia	gag
Tours-Sag38	Singleton	24	Ia	gag
Tours-Sag507	Singleton	24	Ia	gag
Tours-Sag293	23	23	Ia	gag
Tours-P6A1	Singleton	24	Ia	gag
Tours-Sag382	23	23	Ia	gag
Tours-C194	23	220	Ia	gag
Tours-C226	23	23	III	gag
Tours-S165	23	481	III	gag
NEM 316	23	23	III	gag
Tours-S151	23	23	III	gag
Tours-C12	23	391	III	gag
Tours-Sag113	23	366	III	gag
Tours-Sag171	23	1002	III	gag
Tours-Sag456	23	88	Ia	gag
Tours-S14	17	17	III	gag
Tours-S120	17	17	III	gag
Tours-S128	17	17	III	gag
Paris-562	17	17	III	gag
Tours-Sag128	17	17	III	gag
Tours-S94	17	17	III	gag
Tours-S313	17	17	III	gag
Tours-C191	17	17	III	gag
Tours-S202	17	17	III	gag
Tours-Sag299	17	17	III	gag
Tours-Sag463	17	17	III	gag
Paris-509	17	17	III	gag
Tours-Sag545	17	17	III	gag
Tours-S143	17	17	III	gag
Tours-S179	17	17	III	gag
Tours-S2	17	17	III	gag
Paris-498	17	17	III	gag
Tours-S129	17	17	III	gag
Tours-S130	17	17	III	gag
Tours-S135	17	17	III	gag
Tours-S107	17	17	III	gag
Tours-Sag475	17	17	III	gag
Tours-Sag109	17	17	III	gag
Paris-569	17	17	III	tbd
Tours-C213	17	17	III	gag
Tours-C214	17	17	III	gag
Tours-S7	17	17	III	gag
Paris-567	17	17	III	tbd
Tours-S1	17	17	III	gag
Tours-S195	17	17	III	gag
Tours-S192	17	17	III	gag
Paris-533	17	17	III	gag
COH1	17	17	III	gag
BM110	17	17	III	gag
Tours-Sag521	17	17	III	gag
Tours-S103	17	17	III	gag
Tours-Sag492	17	1004	III	gag
Tours-S39	17	17	III	gag
Tours-S191	17	17	III	gag
Tours-C8	17	17	III	gag
Tours-Sag236	17	17	III	gag
Tours-S.ag280	17	17	III	gag
Tours-C38	17	17	III	gag
Paris-598	17	17	III	gag
Paris-447	17	17	III	gag
Tours-Sag234	17	17	III	gag
Paris-429	17	17	III	tbd
Tours-Sag337	17	17	III	gag
Tours-S184	17	17	III	gag
Tours-S194	17	17	III	gag
Tours-S173	17	17	III	gag
Tours-S34	17	17	III	gag
Tours-Sag209	17	17	III	gag
Tours-Sag324	17	17	III	gag
Paris-620	17	291	IV	tbd
Paris-576	17	17	III	gag
Tours-S118	17	17	III	gag
Tours-S24	17	17	III	gag
Tours-C87	singleton	130	V	gag
Tours-Sag117	singleton	130	Ia	gag
Tours-Sag121	singleton	130	Ia	gag

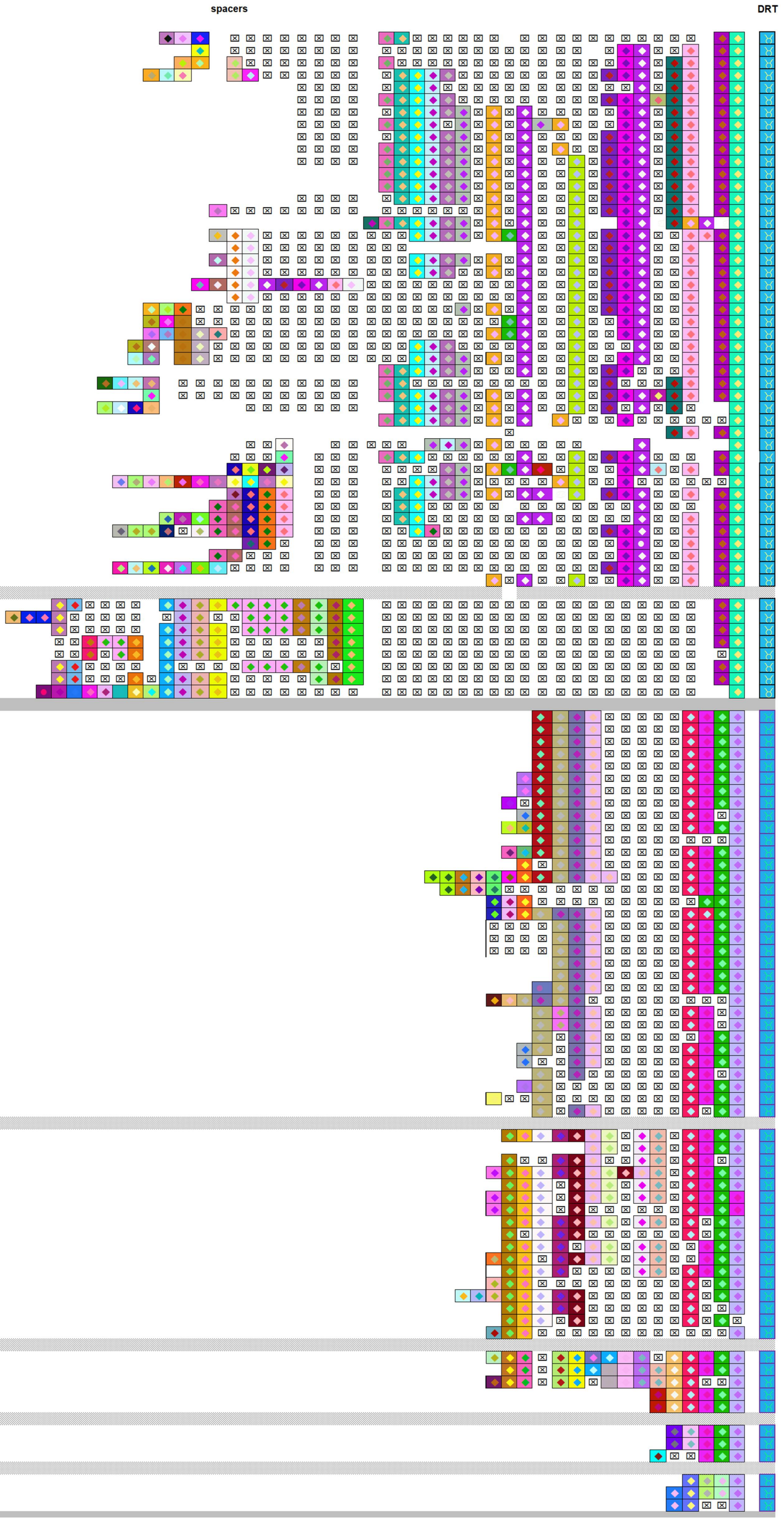

**Tours-S180**	8	8	Ib	:ga
**Tours-S92**	8	8	Ib	:ga
**Tours-C165**	8	8	Ib	:ga
**Tours-C115**	8	8	Ib	:ga
**Tours-P3A1**	8	8	Ib	:ga
**Tours-C175**	8	390	Ib	:ga
**Tours-Sag211**	8	12	II	:ga
**Paris524**	8	8	Ib	:ga
**Tours-C40**	8	8	Ib	:ga
**Tours-Sag498**	8	8	Ib	:ga
**Tours-Sag440**	8	8	V	:ga
**Paris-475**	8	8	Ib	:ga
**Tours-C234**	8	8	Ib	:ga
**Tours-C112**	8	8	Ib	:ga
**Tours-S210**	8	12	Ib	jag
**Tours-C81**	8	12	III	jag
**Tours-Sag488**	8	10	V	:ga
**Tours-P9A1**	singleton	569	II	:ga
**Tours-E207**	8	10	V	:ga
**Tours-Sag499**	8	10	II	:ga
**Tours-Sag142**	8	10	IV	:ga
**Tours-E237**	8	10	Ib	:ga
**Tours-C193**	8	10	IV	ttg
**Paris-493**	8	10	II	agg
**Tours-P2A1**	8	12	Ia	agg
**Tours-Sag247**	8	12	Ib	jag
**Tours-S402**	8	12	Ib	jag
**Tours-S378**	8	12	Ib	jag
**Tours-Sag432**	6	7	V	:ga
**A909**	6	7	Ia	:ga
**Tours-C166**	6	41	III	jag
**Tours-S274**	6	255	Ib	jag
**Tours-Sag286**	6	6	Ib	jag
**Tours-S47**	6	6	Ib	jag
**Tours-Sag357**	6	6	Ib	jag
**Tours-C15**	1	2	II	gtt`
**Tours-C39**	1	2	Ia	jag
**Tours-S.ag138**	1	196	IV	jag
**Tours-S.ag195**	4	3	IV	jag
**Tours-S.ag136**	1	196	IV	jag
**Tours-C219**	1	196	IV	jag
**Tours-C96**	1	196	Ib	jag
**Tours-Sag330**	1	196	IV	jag
**Tours-C188**	1	196	IV	jag
**Paris-614**	1	459	IV	jag
**Tours-Sag45**	1	136	IV	jag
**Tours-Sag371**	1	196	IV	jag
**Paris-437**	1	196	Ia	jag
**Tours-Sag161**	1	1	V	jag
**Tours-Sag153**	1	1	V	jag
**Tours-Sag428**	1	1	V	jag
**Tours-C124**	1	1	V	jag
**Tours-C13**	1	1	V	jag
**Tours-S247**	1	1	V	jag
**Tours-C50**	1	1	II	jag
**Tours-S280**	1	1	V	jag
**Tours-Sag365**	1	1	V	jag
**Tours-C120**	1	1	V	jag
**Tours-C66**	1	1	V	jag
**Tours-C221**	1	1	II	jag
**Tours-S299**	1	1	V	jag
**Tours-P8A2**	1	1	V	jag
**Tours-Sag564**	1	297	V	jag
**Tours-S163**	1	1	V	jag
**Tours-Sag409**	1	1	V	jag
**Tours-Sag169**	1	2	V	jag
**Tours-Sag471**	1	1	V	jag
**Tours-S182**	1	1	V	jag
**Tours-S293**	1	173	V	jag
**Tours-Sag148**	1	1	V	jag
**Tours-Sag443**	1	1	V	jag
**Paris-548**	1	1	V	jag
**Tours-Sag42**	1	1	V	jag
**Tours-C23**	1	1	V	jag
**Tours-Sag249**	1	1	V	jag
**Tours-Sag37**	1	1	V	jag
**Tours-C99**	1	370	V	jag
**Paris-442**	1	1	V	jag
**Tours-Sag544**	1	1005	V	jag
**Tours-Sag459**	1	1	V	jag
**Paris-606**	1	1	V	tbd
**Tours-Sag393**	1	1	V	jag
**Tours-S257**	1	1	V	jag
**Tours-S147**	1	1	V	jag
**Tours-C197**	1	2	Ib	jag
**Tours-Sag140**	1	2	IV	jag
**Tours-Sag228**	1	1	V	jag
**Tours-P10E2**	4	4	Ia	jag
**Tours-S146**	4	4	Ia	jag
**Paris-531**	4	4	Ia	jag
**Tours-Sag305**	4	243	Ia	:ag
**Tours-S352**	19	19	III	jag
**Tours-Sag10**	19	182	III	jag
**Tours-Sag98**	19	28	V	jag
**2603VR**	19	110	V	jag
**Tours-S234**	19	19	III	jag
**Tours-C1**	19	19	III	jag
**Tours-Sag106**	19	28	III	jag
**Tours-C104**	19	19	III	jag
**Tours-C106**	19	19	III	jag
**Tours-S152**	19	19	III	jag
**Tours-Sag278**	19	19	III	jag
**Tours-Sag343**	19	19	III	jag
**Tours-P4E2**	19	19	III	jag
**Tours-Sag477**	19	19	III	jag
**Tours-S127**	19	19	III	jag
**Tours-Sag1**	19	19	III	jag
**Tours-S193**	19	19	III	tag
**Tours-Sag25**	19	19	III	jag
**Tours-S105**	19	19	III	jag
**Tours-Sag400**	19	19	III	jag
**Tours-Sag30**	19	19	III	jag
**Paris-470**	19	19	III	jag
**Paris-476**	19	19	III	jag
**Tours-Sag200**	19	19	V	jag
**Paris-472**	19	19	V	jag
**Tours-Sag396**	19	19	III	jag
**Tours-Sag397**	19	19	III	jag
**Tours-Sag346**	singleton	327	V	jag
**Tours-Sag446**	singleton	327	V	jag
**Tours-Sag485**	19	19	V	jag
**Tours-Sag20**	19	27	III	jag
**Tours-C113**	19	389	III	jag
**Tours-Sag435**	19	19	III	jag
**Tours-Sag259**	19	19	II	jag
**Tours-Sag17**	19	28	II	jag
**Tours-Sag90**	19	28	Ia	jag
**Tours-E546**	19	386	II	agg
**Tours-Sag96**	19	28	II	:tct
**Paris-483**	19	28	II	jag
**Tours-P7A2**	19	28	II	jag
**Paris-503**	19	28	III	jag
**Tours-C97**	19	28	V	jag
**Tours-Sag362**	19	28	II	:ga
**Tours-C94**	singleton	388	V	jag
**Tours-C82**	singleton	388	V	jag
**Tours-Sag536**	singleton	26	V	jag
**Tours-Sag274**	singleton	26	V	jag
**Paris-361**	singleton	26	V	jag
**Tours-Sag43**	singleton	22	II	jag
**Tours-Sag420**	singleton	22	II	jag

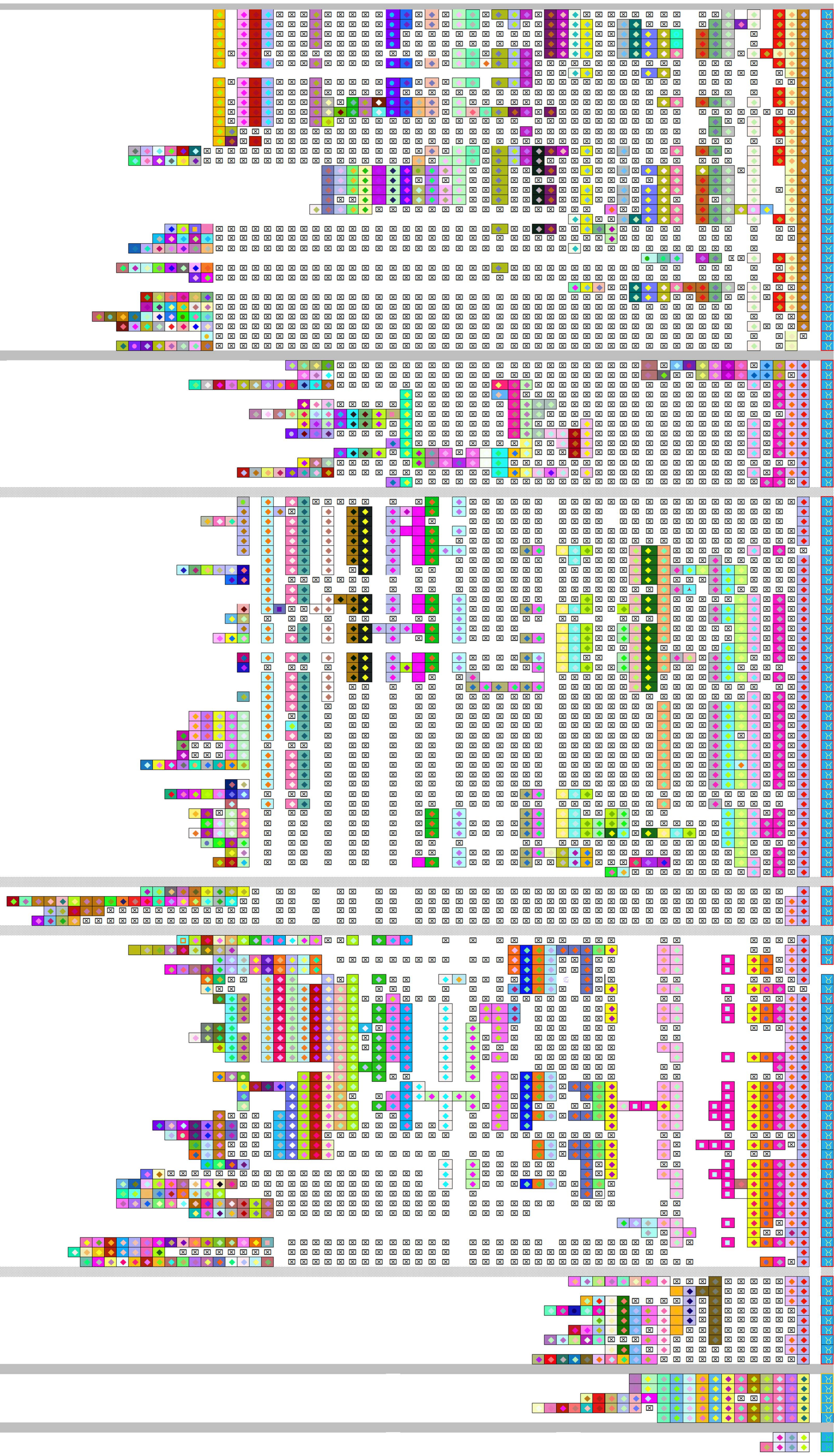

Figure S1. CRISPR1 spacer content of the 255 isolates. The CRISPR1 arrays are represented using a macro-enabled Excel tool, whereby spacers are converted into two-color symbols based on spacer sequence. Gaps (= missing spacers) are shown with a checked boxed symbol ☒ after alignment of identical spacers between strains of the same group. Terminal Direct Repeats (TDRs) are represented using a different color outline according to their sequence. Isolates were distributed according to their CRISPR1 array homology. Isolates were clustered according to their ancestral spacers and TDR composition into 6 clusters (CC23, CC17 – ST130, CC1-CC4, CC19, CC6-CC8, ST26-ST28 and ST22.

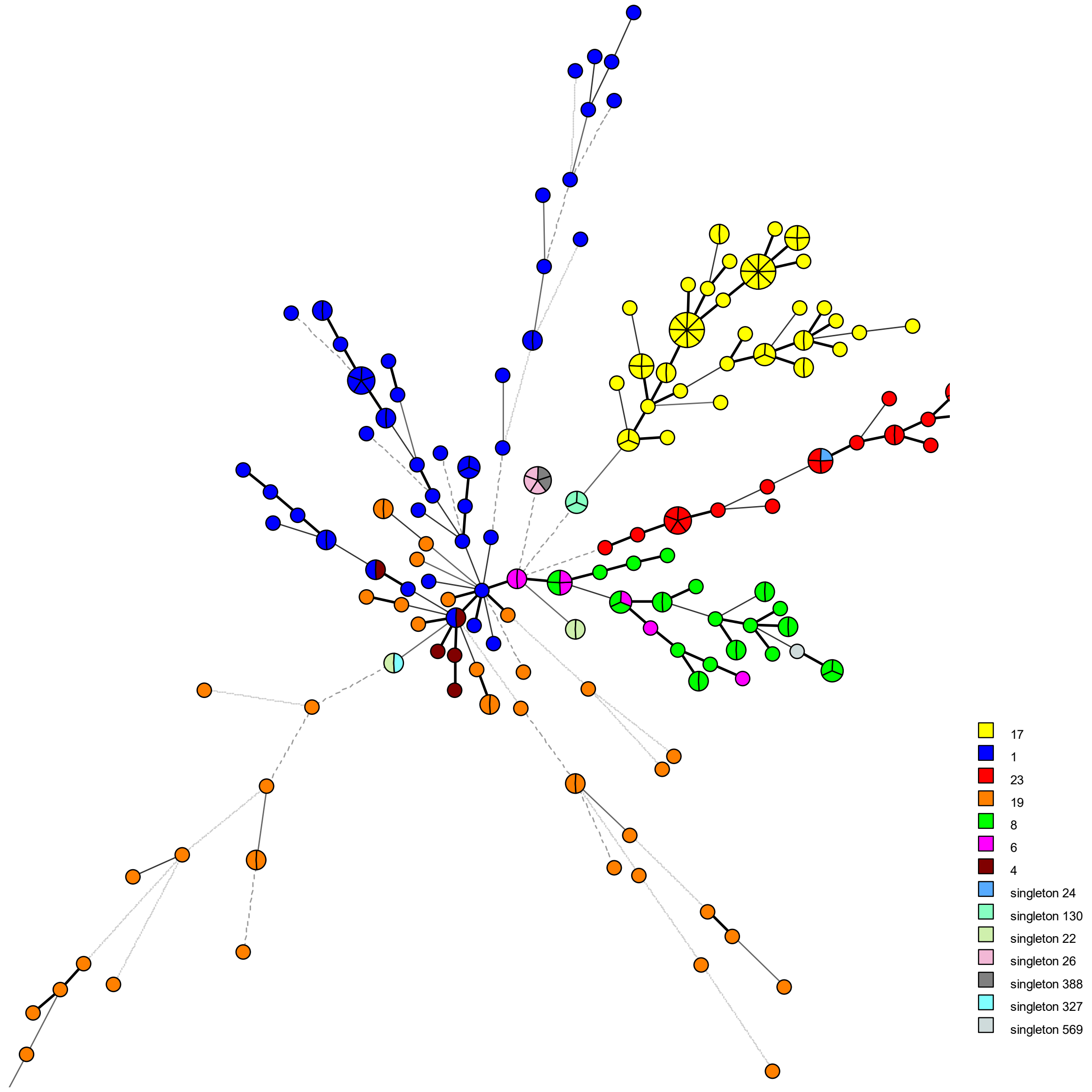

Figure S2. Minimum spanning tree (MST) representation of the 94 CRISPR1 markers clustering scheme. Each circle represents a CRISPR1 genotype and its size is proportional to the number of strains. Each color represents CC or singleton defined by MLST (ex: yellow for CC17). A high level of correlation between this marker selection and MLST type was observed; Circles (representing CRISPR1 genotype) are characterized mostly by a same colour, especially for CC17 and CC23 whereas isolates belonging to CC1 and CC19 are more dispersed.

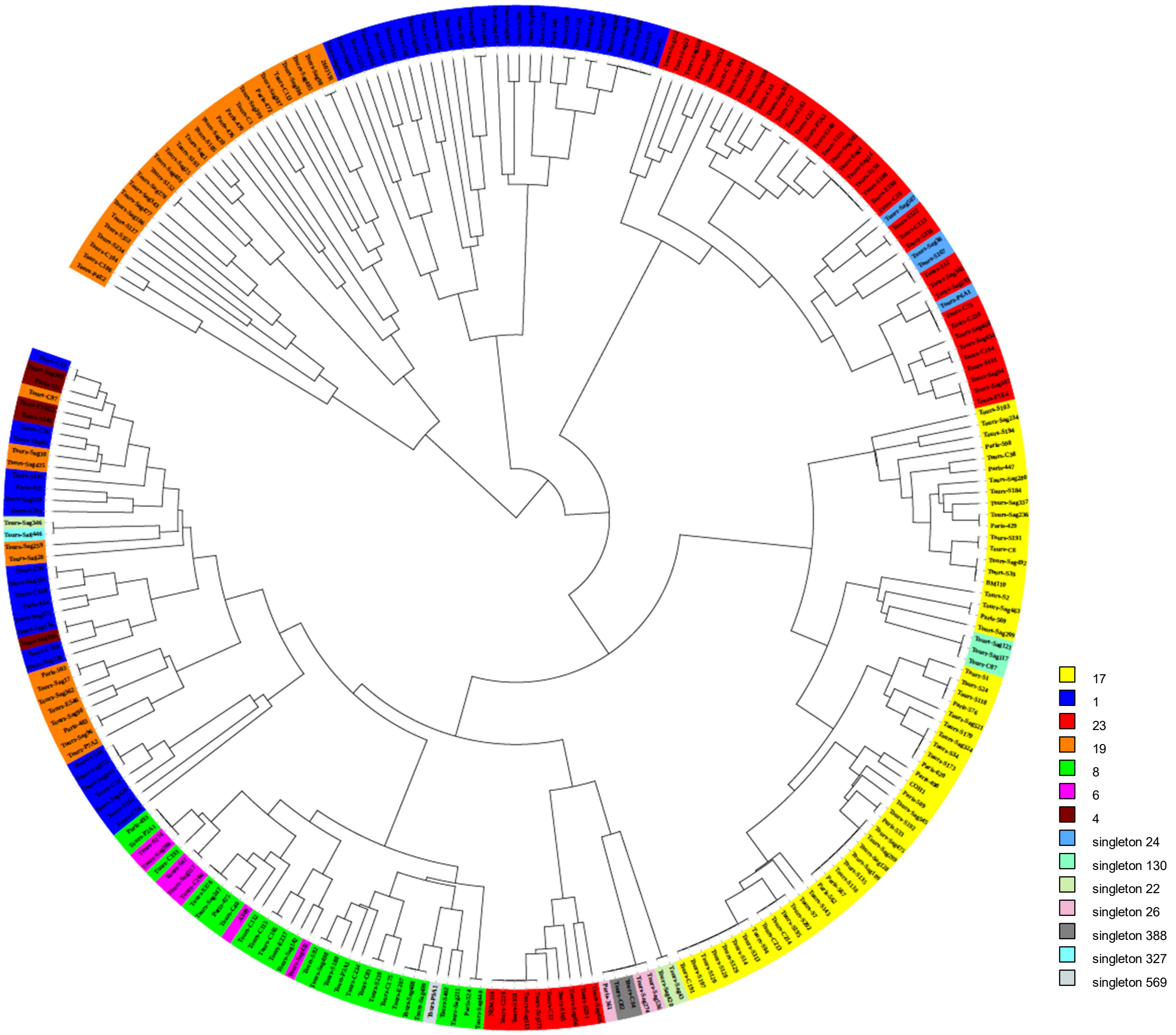

Figure S3. Unweighted pair group (UPGMA) circular tree representation of the 94 CRISPR1 markers clustering scheme. Each color represents CC or singleton defined by MLST (ex: yellow for CC17).

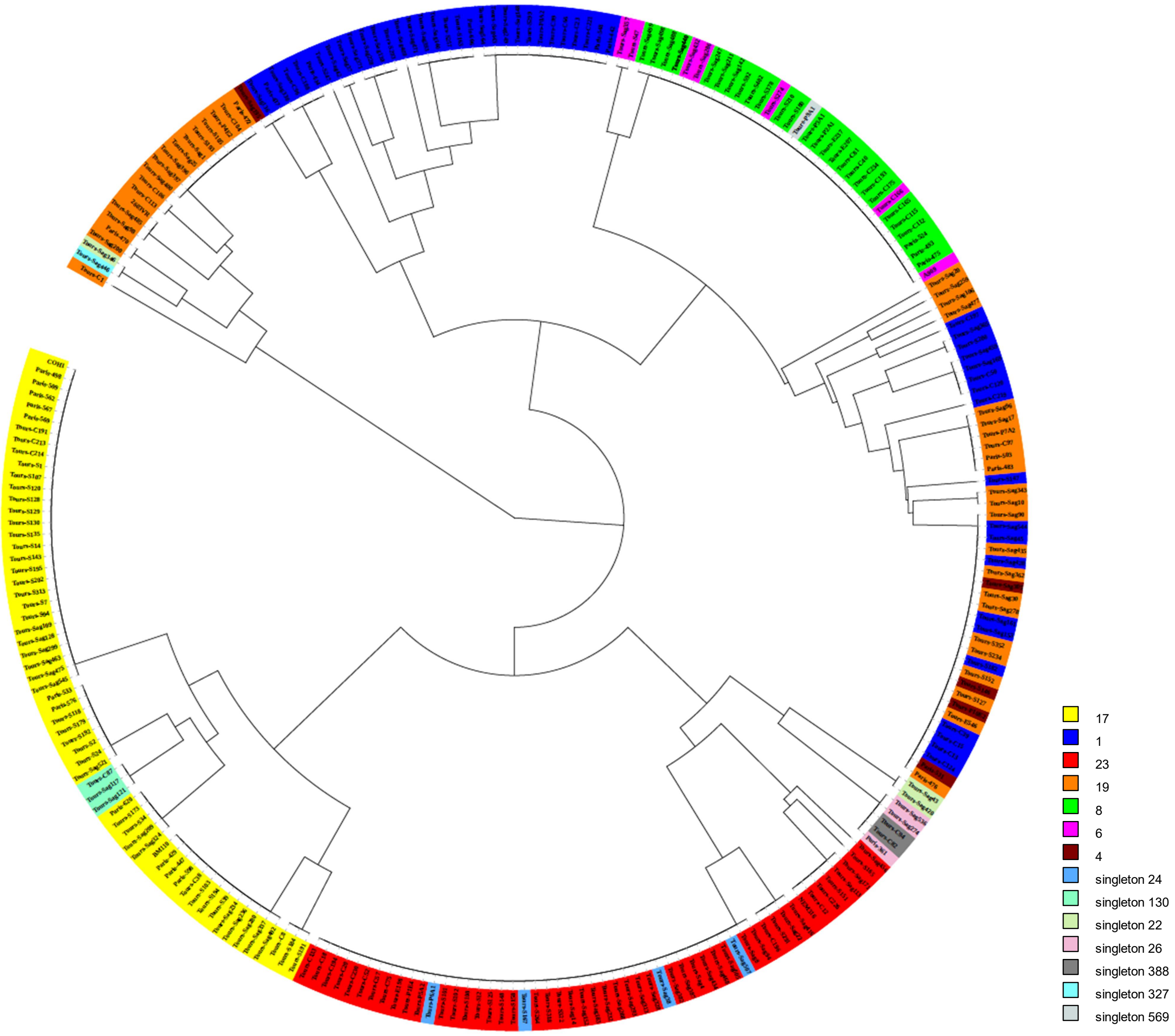

Figure S4. Unweighted pair group (UPGMA) circular tree representation of the 25 CRISPR1 markers clustering scheme. Each color represents CC or singleton defined by MLST (ex: yellow for CC17).

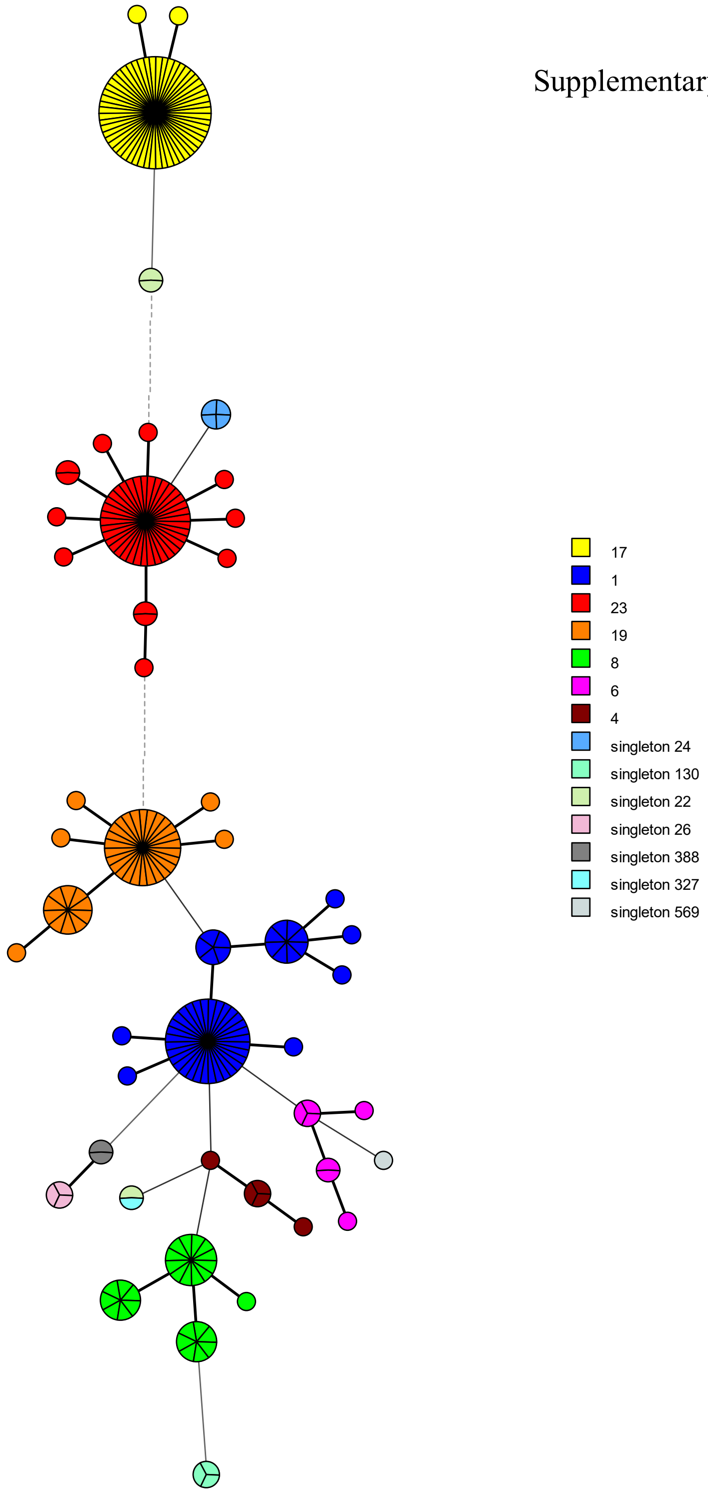

Figure S5. Minimum spanning tree (MST) representation of the clonal complexes defined by eBurst based on MLST data. Each circle represents a ST genotype and its size is proportional to the number of strains. Each color represents CC or singleton defined by MLST (ex: yellow for CC17).

Supplemental tables

Table S1. Primers used for CRISPR1 locus amplification and sequencing

Primer name	Sequence (5' → 3')
CRISPR1 PCR-F	GAGACACAGAGCGACACTATC
CRISPR1 PCR-R	CATTTCTTTCTCCACTATTATAAC
CRISPR1 SEQ-F	GAAGACTCTATGATTTACCGC
CRISPR1 SEQ-R	CAGCAATCACTAAAAGAACCAAC
Sp39F	GCCTTTTCTAACTCTTCAG
Sp40F	TATGTCTTCTAACAGTTGCTTCTTGTGCTT
Sp75F	AGAGTGTGTCCAAGACCAGAGTTACTGTTT
Sp107F	ACCATCAAGGCTCTTATCTGCAGATTGTTA
Sp109F	ACAAAATCTTTTGTTGCTCCTGGACGTATT
Sp154F	GATATTTGGAAAGATTCTGATTTTGGTAAG
Sp177R	AGTTACTGTTGAGGGTAGTCC
Sp246F	GATTAGTTTGCGTACTCGCTC
Sp263F	AAGTGAAGTTGAATTTTATTTGAGATACTA
Sp275F	ACAGACAAAGAAGATGGCAAG
Sp295F	TTGGAAAGATGGCAGAGGGGTTAACGCAGC
Sp298F	CTTTTAACCGTCTCTCCGCTTC
Sp299F	ATGGTCAGAAATCGAAGAAAACGAAGTCGT
Sp305bisF	TTGGAAAAAACACGAAAGTGATATTACTTT
Sp325F	GGATGATTTCGATTATGCGGCGGTGGTTGA
Sp328F	ATTACTTTCAAGATGTCTATGACTATATGC

Sp335F	AAGGGTGTTAGATGATAATACCTTTTTTAA
Sp348F	CGTTCTGCTTTAGTCATATGTGTCCTTTCT
Sp351F	ACAAACCTCTAATGGATAATATAGAACAAA
Sp379F	CATGCCTTTGAAAATAAATTCCGAGCCATT
Sp436F	CCATCCAACCATTTGATTTTGTGTAAATAT
Sp504F	TTTATCCCTAGTAGGTT
Sp511F	GCTTGGGTTTGATAAGGG
Sp531F	AGTTTAGAGTTAACGTCTGTATTTTAGATA
Sp539F	TCTAAGTGCTCGACCATC
Sp650F	ATGCTTGTCAAAGGTAATAAATCTGGAGAT
Sp803F	TCTAATGTATACTGTAACTGAGGCAG
Sp823F	AACACAGCTTCCTCGAAAGGGATATATCTA
Sp830F	TCAAAAGTTTCCACTAATAGCGTTT
Sp856F	TATCAAGTCTGCTTTGTCAACAAAGTCAGC
Sp904bisF	GATATGGTGGACAGGTTCAGCAGGTCATGT
Sp2069F	CCGTGCAAGAGTTAAGGAACTGACACAGCA
Sp2132F	ATGGCTAATTATTGCGTGATGTTAGCGGTT
Sp2166F	TGTTTTTTCTTTCCATATGTGGTGGCTTTC
Sp2191F	AACCTAAAAAGATGTATGAGAAGTTGCCTT
Sp2199F	TCAATATAAATCTGTCCAGTCTTTTTCTCA
Sp2201F	GAAAAATTAAATACTTTCCAATAATTATAA
Sp2211F	AAAATCTAAAAATACATTTTACCGTTTGAA
Sp2228F	ACGTTGAGGGGTTAGTTGCTGCTGGTCTGG

Sp2234F	AGTTTGTTTATGATGAACACCAAAATTTAA
Sp2259F	TAATTTTGTTATATCTATATTTAAGTCTTT
Sp2293F	TTTTCTACTTTATTTCCTACTTCATTTGTT
Sp2312F	CTGTTATATCAACAATTACCAAGTCTGCTG
Sp2325F	ATCGGCAATTTCTTGAAGACGTTCAGCGTT
Sp2359F	AAGGATTTTACTTCAAAGATTTTTTTGTTG
Sp8R	TATCCATCTCGGTGAGATGAGAATTAGCTT
Sp20R	AGATGTCTTGATATCAACTGCTTTAAAAGA
Sp31R	ATCAGTACCAACAAATGATTTTGTACCATC
Sp36R	ACATATCCTTTTGTTAGGTCAAAAGAAGAT
Sp42R	TAAGTAACAAGACAGACTTGAA
Sp52R	TTTATTTTTTCTCAGTTCCTTGATTTTAGA
Sp87R	TATGCAAAGCAGTCACCAGC

Table S2. CRISPR1 direct repeat sequences among *S. agalactiae* isolates. Nucleotide polymorphisms as compared to the typical CRISPR repeat sequence are indicated in red.

Type	Repeat sequence (5'-3')
Typical repeat	GTTTTAGAGCTGTGCTGTTTCGAATGGTTCCAAAAC
Repeat variants	GTTTTAGTGCTGTGCTGTTTCGAATGGTTCCAAAAC<br>GTTTTAGAGCTGTGCTATTTCGAATGGTTCCAAAAC<br>GTTTTAGAGCTGTGTTGTTTCGAATGGTTCCAAAAC<br>GTTTTAAAGCTGTGCTGTTTCGAATGGTTCCAAAAC

Table S3. CRISPR1 marker selection and characteristics of GBS isolates of the study. A). The 94-markers selection included 88 spacers and the five different TDR. (B) The 25-marker selection included the five different TDR and 20 spacers. See the supplementary material file 2.

Table S4. Capsular type repartition of the isolates according to their origin.

Capsular type	Non-Invasive		Invasive		Reference	total
	n	%	strains	%	strains	
Ia	39	71	16	29	1	56
Ib	13	56.5	10	43.5	0	23
II	15	88.2	2	11.8	0	17
III	48	53.3	42	46.7	3	93
IV	10	83.3	2	16.7	0	12
V	39	73.6	14	26.4	1	54
VI	0	0	0	0	0	0
VII	0	0	0	0	0	0
VIII	0	0	0	0	0	0
IX	0	0	0	0	0	0
total	164		86		5	255

Table S5. Clonal complex repartition of the isolates according to their origin**.**

CC	non invasive		invasive		total
	n	%	n	%	
23	34	70.8	14	29.2	48
17	22	39.3	34	60.7	56
1	38	74.5	13	25.5	51
19	31	77.5	9	22.5	40
6	4	66.7	2	33.3	6
8	17	63	10	37	27
4	3	60	2	40	5
singleton 26 /singleton 388	4	80	1	20	5
singleton	11	91.7	1	8.3	12
TOTAL	164		86		250

Table S6. Congruence between CRISPR1 typing, MLST and serotyping (Rand Index)

	**CRISPR1 25 markers**	**CRISPR1 94 markers**	**MLST**	**Capsular typing**
CRISPR1 25 markers	1.000	0.932	0.904	0.797
CRISPR1 94 markers	0,932	1.000	0.902	0.766
MLST	0.904	0.902	1.000	0.840
Serotyping	0.797	0.766	0.840	1.000
